# Class I BASIC PENTACYSTEINE factors regulate *HOMEOBOX* genes involved in meristem size maintenance

**DOI:** 10.1093/jxb/eru003

**Published:** 2014-01-30

**Authors:** Sara Simonini, Martin M. Kater

**Affiliations:** Department of BioSciences, Università degli Studi di Milano, Via Celoria 26, 20133 Milan, Italy

**Keywords:** *Arabidopsis*, BPC, cytokinin, HOMEOBOX, meristem.

## Abstract

The BASIC PENTACYSTEINE (BCP) family is a poorly characterized plant transcription factor family of GAGA BINDING PROTEINS. In *Arabidopsis*, there are seven members (BPC1–7) that are broadly expressed, and they can potentially bind more than 3000 *Arabidopsis* GAGA-repeat-containing genes. To date, BPCs are known to be direct regulators of the *INNER NO OUTER* (*INO*), *SEEDSTICK* (*STK*), and *LEAFY COTYLEDON 2* (*LEC2*) genes. Because of the high functional redundancy, neither single knockout nor double *bpc* mutant combinations cause aberrant phenotypes. The *bpc1-2 bpc2 bpc3* triple mutant shows several pleiotropic developmental defects, including enlargement of the inflorescence meristem and flowers with supernumerary floral organs. Here, we demonstrated through expression analysis and chromatin immunoprecipitation assays that this phenotype is probably due to deregulation of the expression of the *SHOOTMERISTEMLESS* (*STM*) and *BREVIPEDICELLUS/KNAT1* (*BP*) genes, which are both direct targets of BPCs. Moreover, we assigned a role to BPCs in the fine regulation of the cytokinin content in the meristem, as both *ISOPENTENYLTRANSFERASE 7* (*IPT7*) and *ARABIDOPSIS RESPONSE REGULATOR 7* (*ARR7*) genes were shown to be overexpressed in the *bpc1-2 bpc2 bpc3* triple mutant.

## Summary

BPC factors are important regulators of developmental processes. Here we assign a role to BPCs in the control of meristem size, characterizing them as direct regulators of several *HOMEOBOX* genes

## Introduction

The *Arabidopsis* genome contains more than 1900 transcription factor-encoding genes. Based on sequence homology, function, and activity, these factors are subdivided into 64 transcription factor families (Guo *et al.*, 2005). The BASIC PENTACYSTEINE/BARLEY B RECOMBINANT (BPC/BBR) family is a poorly characterized plant-specific transcription factor family. BPC factors might share functional similarity with the Trithorax-like protein named GAGA-associated factor (GAF) of *Drosophila melanogaster*, which transcriptionally regulates expression of the homeotic *HOX* genes and is involved in nucleosome spacing processes (Botas, 1993; Orphanides *et al.*, 1998; Lehmann, 2004; Berger and Dubreucq, 2012). BPC-encoding genes have been identified in different plant species, such as *Glycine max* (soybean), *Hordeum vulgare* (barley), *Oryza sativa* (rice) and *Arabidopsis thaliana* (Sangwan and O’Brian, 2002; Santi *et al.*, 2003; Meister *et al.*, 2004; Kooiker *et al.*, 2005). BPC family members are characterized by the ability to bind the DNA at GA-rich sequences: the GAGA BINDING PROTEIN (GBP) of soybean specifically binds a (GA)_9_ repeat sequence located in the *Glutamate 1-Semialdehyde Aminotransferase* (*Gsa1*) gene promoter (Sangwan and O’Brian, 2002), the BARLEY B RECOMBINANT (BBR) factor binds (GA)_8_ sequences *in vitro* (Santi *et al.*, 2003), and the *Arabidopsis* BPC proteins specifically recognize (GA)_6_ and (GA)_9_ repeats *in vitro* and *in vivo* (Meister *et al.*, 2004; Kooiker *et al.*, 2005; Simonini *et al.*, 2012).

The seven BPCs encoded by the *Arabidopsis* genome sequence are divided into three classes, namely class I (*BPC1–3*), class II (*BPC4–6*), and class III (*BPC7*). Except for *BPC5*, which is thought to be a pseudogene, they are all ubiquitously expressed transcriptional activators and repressors (Meister *et al.*, 2004; Monfared *et al.*, 2011).

More than 3000 *Arabidopsis* genes contain at least one GA-rich stretch in their regulatory region, and combining multiple *bpc* mutant alleles together results in a broad range of developmental defects (Meister *et al.*, 2004; Monfared *et al.*, 2011), suggesting that the function of BPCs are not specific for one developmental process and/or tissue. For instance, BPCs are known to be regulators of YABBI transcription factors, such as *INNER NO OUTER* (*INO*), a gene involved in ovule development (Meister *et al.*, 2004). BPCs are also involved in seed development, being regulators of the B3-domain *LEAFY COTYLEDON 2* gene (*LEC2*; Berger *et al.*, 2011). Moreover BPCs regulate the expression of the ovule identity MADS-domain transcription factor encoding gene *SEEDSTICK* (*STK*; Pinyopich *et al.*, 2003; Favaro *et al.*, 2003) by looping its regulatory region and through an interaction with a MADS-domain transcription factor containing repressor complex (Kooiker *et al.*, 2005; Simonini *et al.*, 2012).

In 2003, Santi and colleagues demonstrated that, in barley, the BBR factor directly regulates transcription of the HOMEOBOX transcription factor *BKN3*. The orthologue of *BNK3* in *Arabidopsis* is named *SHOOTMERISTEMLESS* (*STM*), and is strongly expressed in meristematic tissues where it is necessary for setting up and maintaining the meristem (Endrizzi *et al.*, 1996; Long *et al.*, 1996). *STM* promotes cytokinin (CK) synthesis, a class of plant hormones involved in the maintenance of meristem identity, size, and activity (Jasinski *et al.*, 2005; Leibfried *et al.*, 2005; Yanai *et al.*, 2005; Bartrina *et al.*, 2011). Plants with hyperproduction or slow degradation of CK display compact inflorescences, extra floral organs, and altered phyllotaxis caused by an enlarged and overproductive inflorescence meristem (IM) (Venglat and Sawhney, 1996; Bartrina *et al.*, 2011; Bencivenga *et al.*, 2012).

Here, we unravelled the role of class I BPCs in the control of meristem size, characterizing them as direct regulators of several HOMEOBOX genes, such as *STM* and *KNOTTED-LIKE FROM ARABIDOPSIS THALIANA* (*KNAT*) genes like *BREVIPEDICELLUS/KNAT1* (*BP*). Moreover, we linked the *bpc1-2 bpc2 bpc*3 triple mutant IM phenotype to increased CK synthesis in the IM.

## Materials and methods

### Plant material and growth conditions

The *A. thaliana* ecotype used in this work was Col-0; plants were grown under short-day conditions for 2 weeks (22 °C, 8h light/16h dark) and then moved to long-day conditions (22 °C, 16h light/8h dark). The *bpc1-2 bpc2 bpc3* triple mutant was kindly provided by Professor C. Gasser. The *pBP::GUS* and the *pCLV3::GUS* lines were obtained from the Nottingham Arabidopsis Stock Centre.

### *In situ* hybridization and β-glucuronidase (GUS) staining

*In situ* hybridization experiments were performed as described previously by Dreni *et al.* (2011). The *STM* antisense probe was prepared according to the method of Long *et al.* (1996) and the *ARR7* probe according to the method of Buechel *et al.* (2010). GUS staining was performed as described by Simonini *et al.* (2012).

### Plasmid construction and ethanol induction experiments

The EAR motif was added at the C terminus of the *BPC1*-coding sequence (see primer sequences in Supplementary Table S2 available at *JXB* online). The fragment was cloned into the pB2GW7 plasmid (35S) and the binary pFLUAR (pAlc) vector carrying DsRed as visual selection markers (Battaglia *et al.*, 2006) passing through the pENTRY-D-TOPO vector (Life technologies). *Arabidopsis* plants were transformed by the floral-dip method (Clough and Bent, 1998).

The *35S::BPC1-EAR* lines were selected by BASTA treatment whereas the seeds of the pAlc-BPC1-EAR motif were selected under a Leica MZ FLIII stereomicroscope and immediately transferred on soil. The *pALC::BPC1-EAR* plants were inducted for 4–6 d for 8h per day using ethanol vapour, which was applied at bolting. Inflorescences were collected at 4 and 6 d of induction.

### RNA isolation, reverse transcription-PCR and quantitative real-time PCR (qRT-PCR) analysis

Total RNA was extracted from young inflorescences (meristem, floral buds, and young flowers) using the LiCl method (Verwoerd *et al.*, 1989) for all expression analyses (*STM*, *BP*, *IPT7*, and *WUS*). Total RNA was treated with an Ambion TURBO DNA-free DNase kit and then reverse transcribed using an ImProm-II™ Reverse Transcription System (Promega). The cDNAs were standardized relative to *UBIQUITIN10* (*UBI10*) and *PROTEIN PHOSPHATASE 2A SUBUNIT A3* (*PP2A*; At1g13320) transcripts, and gene expression analyses were performed using an iQ5 Multi Colour Real-Time PCR detection system (Bio-Rad) with a SYBR Green PCR Master Mix (Bio-Rad). Baseline and threshold levels were set according to the manufacturer’s instructions.

For reverse transcription-PCR and qRT-PCR primers, see Supplementary Table S2 available at *JXB* online.

### Chomatin immunoprecipitation (ChIP) assays

ChIP experiments were performed as reported previously using a polyclonal antibody raised against the entire BPC1 protein (Simonini *et al.*, 2012). Chromatin was extracted from wild-type plant (Col-0) inflorescences and from the *bpc1-2 bpc2 bpc3* triple mutant, which was used as negative control. The DNA fragments obtained from the immunoprecipitated chromatin were amplified by qRT-PCR using specific primers (see Supplementary Table S2 available at *JXB* online). Three real-time PCR amplifications were performed for three independent chromatin extractions. For the complete primer sets see Supplementary Table S2 available at *JXB* online. Enrichment of the target region was determined using an iQ5 Multi Colour Real-Time PCR detection system (Bio-Rad) with a SYBR Green PCR Master Mix (Bio-Rad). The qRT-PCR assays and the fold enrichment calculations were performed as described by Gregis *et al.* (2008).

### Optical, confocal, and scanning electron microscopy.

Samples for GUS and *in situ* hybridization analyses were imaged using a Zeiss Axiophot D1 microscope (http://www.zeiss.com/) equipped with differential interface contrast optics. Images were captured on an Axiocam MRc5 camera (Zeiss) using the AXIOVISION program (version 4.4).

Propidium iodide staining was performed as described by Truernit *et al.* (2008). Samples were imaged with an SP5 Leica confocal microscope. Images were subsequently analysed with Fiji software (Schindelin *et al.*, 2012).

Scanning electron microscopy (SEM) analysis was performed according to the method of Grandi *et al.* (2012).

### Accession numbers

Details of accession numbers are as follows: *BPC1*, AT2G01930; *BPC2*, AT1G14685; *BPC3*, AT1G68120; *STM*, AT1G62360; *BP*, AT4G08150; *KNAT4*, AT5G11060; *KNAT5*, AT4G32040; *KNAT6*, AT1G23380; *KNAT7*, AT1G62990; *WUS*, AT2G17950; *WOX3*, AT2G28610; *WOX9*, AT2G33880; *RPL*, AT5G02030; *BLH1*, AT2G35940; *CRN*, AT1G52150; *CLV3*, AT2G27250; *ARR7*, AT1G19050; *IPT7*, AT3G23630.

## Results

### class I BPCs regulate inflorescence and flower development

Phenotypic analysis of the *Arabidopsis bpc1-2*, *bpc2*, and *bpc3* single mutants or double mutant combinations did not show any obvious developmental defect, probably due to the functional redundancy among the different *BPC* genes (Monfared *et al.*, 2011). However, when the three mutants were combined in the *bpc1-2 bpc2 bpc3* triple mutant, there were not only defects in reproductive organs (which also affect plant fertility; Monfared *et al.*, 2011) but also evident developmental aberrations in the structure and organization of the inflorescence and flowers.

**Fig. 1.**
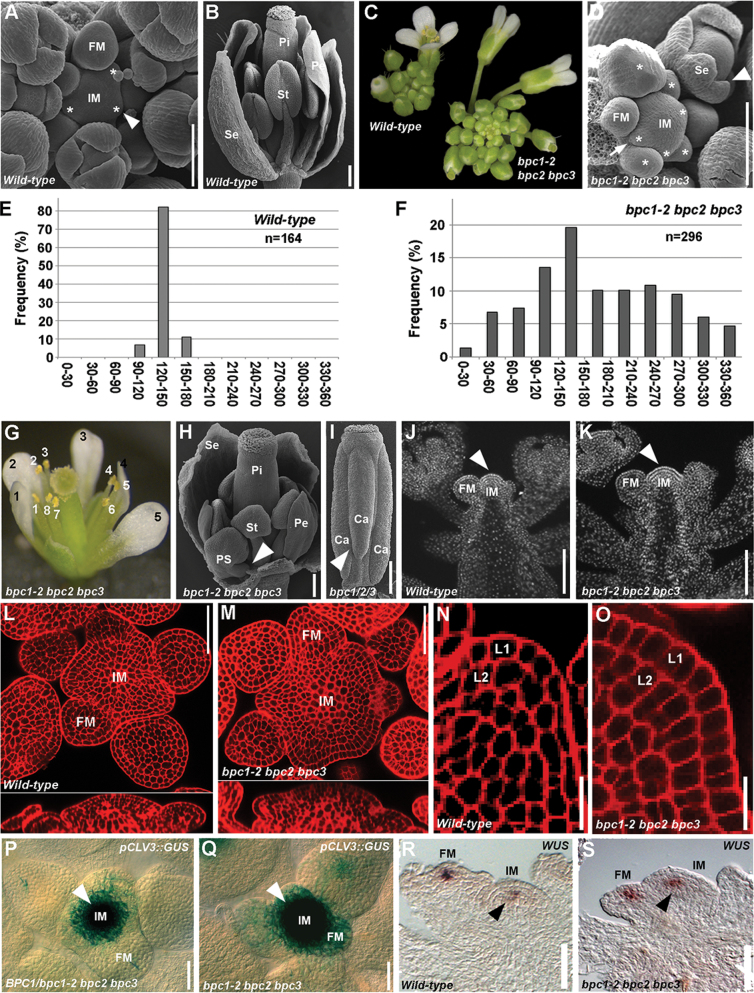
The *bpc1-2 bpc2 bpc3* mutant has enlarged IMs. (A) SEM of a wild-type inflorescence apex. Asterisks indicate developing FMs. (B) SEM of wild-type flower. One sepal has been removed. (C) A *bpc1-2 bpc2 bpc3* inflorescence (right) compared a wild-type inflorescence (left). (D) SEM of a *bpc1-2 bpc2 bpc3* inflorescence apex showing the IM with several developing floral primordia (arrow). Young floral buds presented fused extra sepals (arrowhead). Asterisks indicate developing FMs. (E) Frequency of divergence angle of siliques in wild-type plants: the majority of the angles fell in the 120–150° class. (F) Frequency of divergence angle of siliques in *bpc1-2 bpc2 bpc3* mutant plants: the siliques were randomly distributed along the stem. (G) A *bpc1-2 bpc2 bpc3* mutant flower displaying eight stamens (white numbers) and five petals (black numbers). (H) SEM of a *bpc1-2 bpc2 bpc3* mutant flower. One sepal has been removed to reveal a petaloid extra stamen developing from the second whorl (arrowhead). (I) SEM of a *bpc1-2 bpc2 bpc3* pistil with a third fused carpel (arrowhead). (J) DAPI staining of a longitudinal section of a wild-type inflorescence. The arrowhead indicates the IM. (K) DAPI staining of a longitudinal section of a *bpc1-2 bpc2 bpc3* inflorescence. Compare the IM (arrowhead) size with the wild-type one in (J). (L, M) mPS-PI staining of a wild-type (L) and *bpc1-2 bpc2 bpc3* (M) inflorescence apex in transversal (upper panel) and longitudinal (lower panel) sections. Note the increased meristem dimension in the *bpc1-2 bpc2 bpc3* triple mutant. (N, O) Magnification of mPS-PI staining of the L1 and L2 layers of an inflorescence apex of a wild-type (N) and *bpc1-2 bpc2 bpc3* triple mutant (O): the cells of the *bpc1-2 bpc2 bpc3* mutant were slightly bigger than those of the wild type. (P) Expression of *pCLV3::GUS* in the *BPC1/bpc1-2 bpc2 bpc3* background (plant does not present the *bpc1-2 bpc2 bpc3* phenotype). The arrowhead indicates the IM. (Q) Expression of *pCLV3::GUS* in the *bpc1-2 bpc2 bpc3* background. Note the increase of GUS expression at the IM (arrowhead) compared with that in (L). (R) *In situ* hybridization with a *WUS*-specific antisense (as) probe in wild-type IMs (arrowhead) and FMs. (S) *In situ* hybridization with *WUS* specific antisense (as) probe in the *bpc1-2 bpc2 bpc3* IM (arrowhead) and FMs. Ca, carpel; Pe, petal; Pi, pistil; PS, petaloid stamen; Se, sepal; St, stamen. Bars, 100 µm. (This figure is available in colour at *JXB* online.)

A wild-type *Arabidopsis* IM is a symmetrical dome-shaped structure that produces floral meristems (FMs) in a spiral phyllotaxy at distances of 137.5°. Typically, three FMs that have not yet developed floral organs can be observed (Fig. 1A). From the FM, four types of floral organs develop in concentric whorls. From the outer to the inner whorl the following develop: four sepals, four petals, six stamens, and a pistil composed of two fused carpels (Fig. 1B). Careful phenotypic analysis of the *bpc1-2 bpc2 bpc3* triple mutant showed that the inflorescence developed more flowers than the wild-type plant and that they seemed to be randomly positioned (Fig. 1C). SEM analysis showed that more FMs developed: four or more FMs could be detected at the same time, and these were randomly positioned on the IM surface (Fig. 1D).

The characterization of the phyllotactic pattern of the flowers by measuring the divergence angle between successive siliques along the main inflorescence stem (Peaucelle *et al.*, 2007; Pinon *et al.*, 2013) further indicated the random positioning of flowers. In wild-type plants, the majority of the angles fell into the 120–150° class (which contain the theoretical angle 137.5°; Fig. 1E), whereas in the *bpc1-2 bpc2 bpc3* mutant, the siliques were randomly distributed along the stem (Fig. 1F and Supplementary Fig. S1 available at *JXB* online) and extreme distributions were frequently observed (i.e. angles in the 30–60° and 300–330° classes).

Moreover, more than 90% of the *bpc1-2 bpc2 bpc3* mutant flowers were composed of five or more sepals, which were often fused along their margins, five or more petals, eight or more stamens (which sometimes arose from the second whorl and presented petaloid features), and up to three carpels (Fig. 1G–I).

As a similar phenotype was observed in plants with hyperproliferative IM tissue (Laufs *et al.*, 1998), the size of the *bpc1-2 bpc2 bpc3* IM was analysed by 4′,6-diamidino-2-phenylindole (DAPI) staining and compared with that of the wild-type (Fig. 1J, K). This analysis revealed that the *bpc1-2 bpc2 bpc3* triple mutant IM was significantly enlarged when compared with that of the wild-type. Detailed morphological analyses of the inflorescence apex by modified pseudo-Schiff propidium iodide (mPS-PI) staining (Truernit *et al.*, 2008) confirmed that the *bpc1-2 bpc2 bpc3* IM was larger than that in wild-type plants, being more expanded and rounded (Fig. 1L, M). The enlargement of the meristem could be a consequence of an increase in cell proliferation and/or cell size. The cells of the L1 and L2 layers of the *bpc1-2 bpc2 bpc3* IM were clearly increased in size and had a more rectangular shape with respect to that of the wild-type (Fig. 1N, O). Although the phenotype suggested that cell numbers were increased, a more detailed analysis is needed to confirm this. To obtain further support for an increase in size and activity of the meristem, we investigated the expression of *CLAVATA3* (*CLV3*) and *WUSCHEL* (*WUS*) in the *bpc1-2 bpc2 bpc3* triple mutant, as an increase in their expression domain has shown to be indicative of a larger meristem (Clark *et al.*, 1995; Schoof *et al.*, 2000). The *pCLV3::GUS* reporter construct (Gross-Hardt *et al.*, 2002) was introduced in the *bpc1-2 bpc2 bpc3* triple mutant background. GUS assays showed that, in triple mutant plants, *CLV3* expression was stronger and more expanded when compared with that in the heterozygous mutant plants belonging to the same segregating population (Fig. 1P, Q). To investigate *WUS* expression in the inflorescence, we performed *in situ* hybridization analysis using a *WUS*-specific probe (Brambilla *et al.*, 2007). This analysis showed that, in the triple mutant, the expression domain of *WUS* was both in the IMs and FMs similar to that observed in the wild type (Fig. 1R, S, and Supplementary Fig. S2 available at *JXB* online).

Taken together, these data suggested that BPC proteins of class I are involved in regulating IM and FM size by the negative control of meristem activity.

### BPC1 is involved in many aspects of plant development

**Fig. 2.**
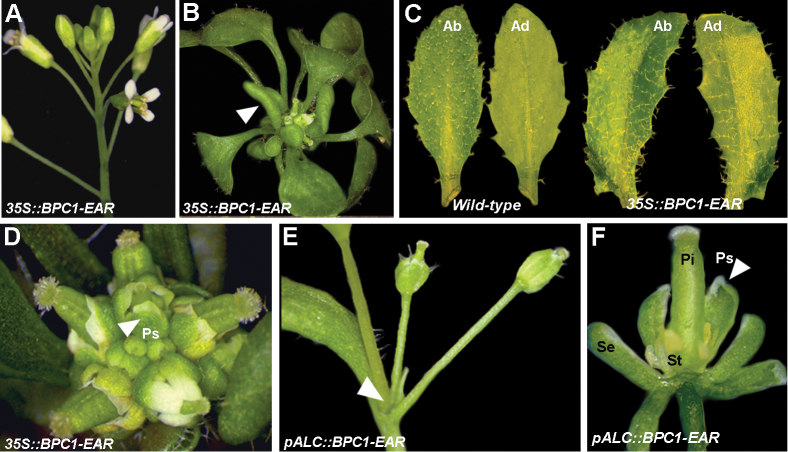
Expression of the BPC1–EAR chimeric protein causes strong developmental defects. (A) A *35S::BPC1–EAR* plant with wild-type phenotype. (B) A *35S::BPC1–EAR* plant with a severe phenotype. Leaves are small and curled (arrowhead). (C) Leaf of a *35S::BPC1–EAR* plants with both abaxial and adaxial sides covered by trichomes. (D) Inflorescence of a *35S::BPC1–EAR* plant with a severe phenotype. Flowers displayed petaloid sepals (arrowhead). (E) A *pALC::BPC1–EAR* plant after 6 d of ethanol induction in which the IM arrested prematurely (arrowhead). (F) A flower of a *pALC::BPC1–EAR* plant after 4 d of ethanol induction with petaloid sepals (arrowhead), no petals, and short and aberrant stamens. Pi, pistil; Se, sepal; St, stamen; Ps, petaloid sepals; Up, upper side; Lo, lower side. (This figure is available in colour at *JXB* online.)

The BPCs are transcriptional regulators that are thought to function both as activators and repressors of gene expression (Meister *et al.*, 2004; Kooiker *et al.*, 2005; Berger and Dubreucq, 2012; Simonini *et al.*, 2012). To investigate the regulatory potential of these factors in more detail, we fused BPC1 to the strong EAR repressor domain (Hiratsu *et al.*, 2003). The *BPC1–EAR* chimeric open reading frame was placed under the control of the 35S cauliflower mosaic virus promoter and introduced into wild-type *Arabidopsis* plants. Of the 270 transformants, 90% (*n*=239) did not show any phenotype, being completely indistinguishable from the wild type (Fig. 2A), whereas the remaining 10% of plants (*n*=31), which showed the highest expression of the transgene (Supplementary Fig. S3 available at *JXB* online), exhibited severe defects during vegetative and reproductive development. These *35S::BPC1–EAR* plants had few small curved leaves, which never reached the wild-type size (Fig. 2B).

Analysis of the number of trichomes on the adaxial side of the leaf is a useful criterion for understanding whether a leaf has (partially) lost its adaxial/abaxial symmetry identity. During *Arabidopsis* leaf development, the first two to three leaves lack trichomes on their adaxial (lower) surface. Later during development, trichomes can be observed on both sides of the leaf but are most abundant on the abaxial (upper) surface (Larkin *et al.*, 1996). Analysis of the fifth developing leaf suggested that the *35S::BPC1–EAR* leaves partially lacked adaxial/abaxial identity, as a conspicuous number of trichomes was present on the adaxial side of the leaf (Fig. 2B, C). This features was also present in most of the curved leaves of the *35S::BPC1–EAR* rosette.

Furthermore, the plants developed a compact and disorganized inflorescence, bearing aberrant flowers, which remained attached to the rosette due to the inability to develop a stem (Fig. 2D). To avoid the strong pleiotropic phenotypes observed in the *35S::BPC1–EAR* lines during vegetative growth and to be able to investigate better the role of *BPC1* during flower development, the chimeric *BPC1–EAR* fusion gene was placed under the control of the *AlcR/AlcA* ethanol-inducible promoter system (Roslan *et al.*, 2001). Ten wild-type plants and 18 *pAlcA::BPC1–EAR* plants were treated with ethanol vapour for 8h d^–1^ for 4 and 6 d, consecutively. The treatment was applied when the plants had switched from the vegetative to the reproductive phase, and a small cluster of floral buds was visible at the centre of the basal rosette. Whereas the wild-type plants treated with ethanol vapour showed no altered phenotype (data not shown), all 18 *pAlcA::BPC1–EAR* plants showed a strong phenotype when treated with ethanol. In these plants, the inflorescence was composed of only a few flowers, probably due to a premature arrest of IM activity (Fig. 2E). Moreover, the flowers were aberrant and sterile, and the perianth organs were composed only of sepals of which some had petaloid features (Fig. 2F).

The severe pleiotropic phenotypes observed in these BPC1–EAR plants during vegetative and reproductive growth suggested that this factor is involved in many different developmental processes, including meristem activity.

### *The KNOX* genes *STM* and *BP* are direct targets of BPCs of class I

**Fig. 3.**
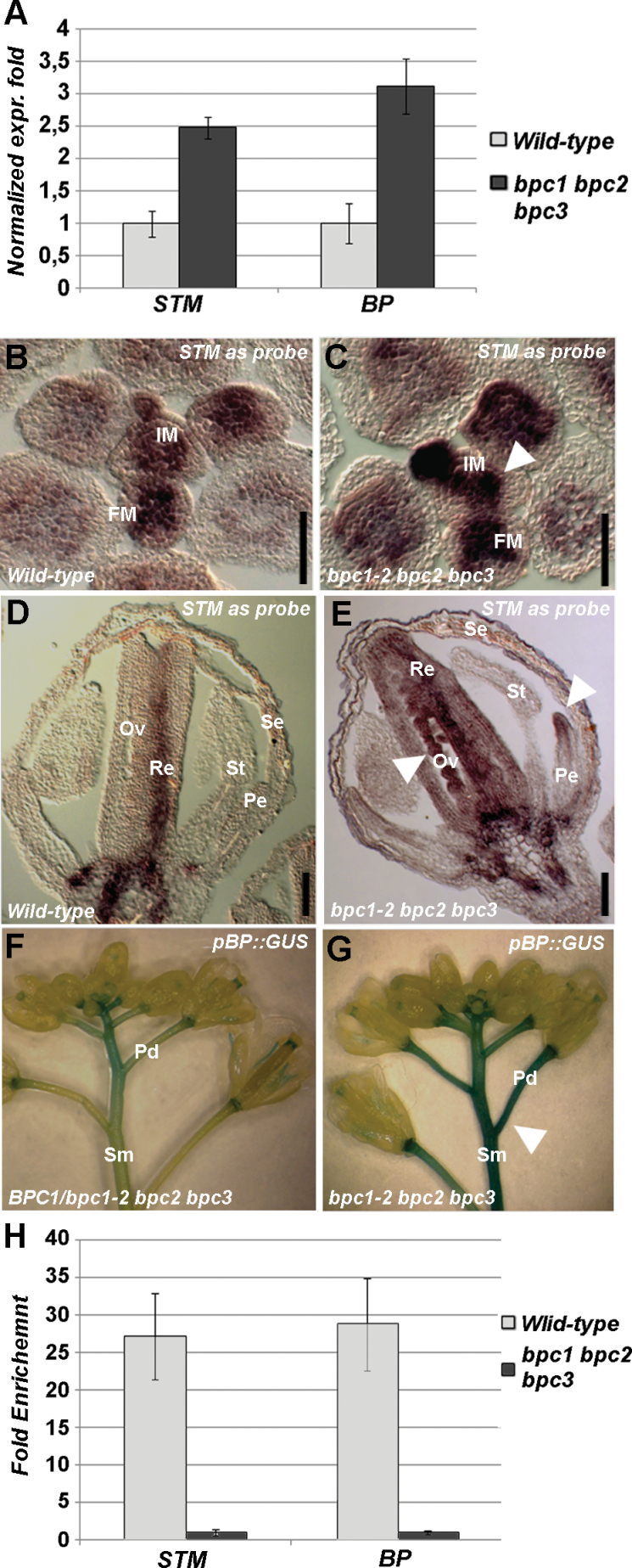
STM and BP are direct targets of class I BPCs. (A) qRT-PCR of the wild type and *bpc1-2 bpc2 bpc3* triple mutant to determine *STM* and *BP* expression levels. (B–E) *In situ* hybridization using an *STM*-specific antisense probe of transversal sections of (B, D) and wild-type (C, E) *bpc1-2 bpc2 bpc3* inflorescences. The signal was more intense and expanded in the meristems of the mutant (arrowhead in B). In the wild-type flower, the signal was localized in the replum and at the base of the flower, whereas in the triple mutant it was also in the petal and ovules (arrowheads in E). (F, G) GUS staining of *BPC1/bpc1-2 bpc2 bpc3* (F) and *bpc1-2 bpc2 bpc3* (G) inflorescences of plants containing the *pBP::GUS* construct. In the homozygous triple mutant, the signal was expanded and more persistent in both the pedicels and stems (arrowhead in G). (H) ChIP analysis revealing that BPCs of class I directly bind the *STM* and *BP* promoter; the *bpc1-2 bpc2 bpc3* was used as a negative control. Each bar shows the average of three independent ChIP experiments (±standard deviation). Pe, petals; Se, sepals; St, stamen; Re, replum; Ov, ovules; Sm, stem; Pd, pedicel. Bars, 50 µm. (This figure is available in colour at *JXB* online.)

The *HOMEOBOX* gene family is large in *Arabidopsis* and can be subdivided in different subfamilies (Chan *et al.*, 1998; Ariel *et al.*, 2007). *The KNOTTED1-like homeobox* (*KNOX*) family includes several genes essential for meristem maintenance and floral organ development such as *STM*, *BP* and *KNAT2–7*. As *STM* and *BP* are important for meristem maintenance and inflorescence architecture (Long *et al.*, 1996; Venglat *et al.*, 2002), the expression levels of *STM* and *BP* were investigated by qRT-PCR of wild-type and *bpc1-2 bpc2 bpc3* triple mutant inflorescences. Both *STM* and *BP* expression levels were higher (2.5-fold more for *STM* and 3.2-fold more for *BP*) in the *bpc1-2 bpc2 bpc3* triple mutant than in the wild-type (Fig. 3A), suggesting that BPCs of class I act as repressors of *STM* and *BP*.

*In situ* hybridization using an *STM*-specific antisense probe (Long *et al.*, 1996) revealed that its expression domain seemed to be enlarged in the *bpc1-2 bpc2 bpc3* triple mutant IM when compared with that of the wild-type, which is probably due to enlargement of the meristem as observed in the triple mutant (Fig. 3B, C). Later during flower development, *STM* expression was detected ectopically in *bpc1-2 bpc2 bpc3* triple mutant petals and ovules (Fig. 3D, E), further supporting the hypothesis that BPCs are repressors of *STM*.

To analyse temporal and spatial *BP* expression in the *bpc1-2 bpc2 bpc3* triple mutant background, we introduced in this mutant the *BP::GUS* reporter construct (Ori *et al.*, 2000). GUS assays showed that *BP* expression was stronger and more persistent, and was expanded throughout the stem and pedicels of the *bpc1-2 bpc2 bpc3* triple mutant plants when compared with the segregating genotypes belonging to the same population (Fig. 3F, G). This was in agreement with the upregulation of *BP* expression detected by RT-PCR and strengthened the hypothesis that BPCs of class I act as repressors of *BP* transcription.

Analysis of the promoter regions of *STM* and *BP* revealed that they contained GA-rich sequences that differed from the GA-rich consensus sequences found in the promoter of the MADS-box gene *STK* (Kooiker *et al.*, 2005). The latter are relatively short in sequence (9–15bp) and distributed along the 2900bp of the *STK* regulatory region, whereas the GA repeats located in the *STM* and *BP* promoters were extremely long (up to 50bp), unique, and located within 500bp of the transcription start site (Supplementary Fig. S4 available at *JXB* online). The ability of class I BPCs to bind these GA-rich sequences was tested in three independent ChIP assays (Fig. 3H) using chromatin extracted from wild-type inflorescences and a polyclonal antibody that recognized BPCs of class I (Simonini *et al.*, 2012). Chromatin extracted from inflorescences of the *bpc1-2 bpc2 bpc3* triple mutant was used as a negative control, and as a positive control the *STK* promoter was used (results not shown; Simonini *et al.*, 2012). In all three biological ChIP replicates, the GA-rich stretches in both the *STM* and *BP* promoters were strongly enriched (Fig. 3H), confirming that, in *Arabidopsis* inflorescences, the class I BPCs directly bind and regulate the expression of *STM* and *BP*.

### *HOMEOBOX* genes are direct target of BPCs

**Fig. 4.**
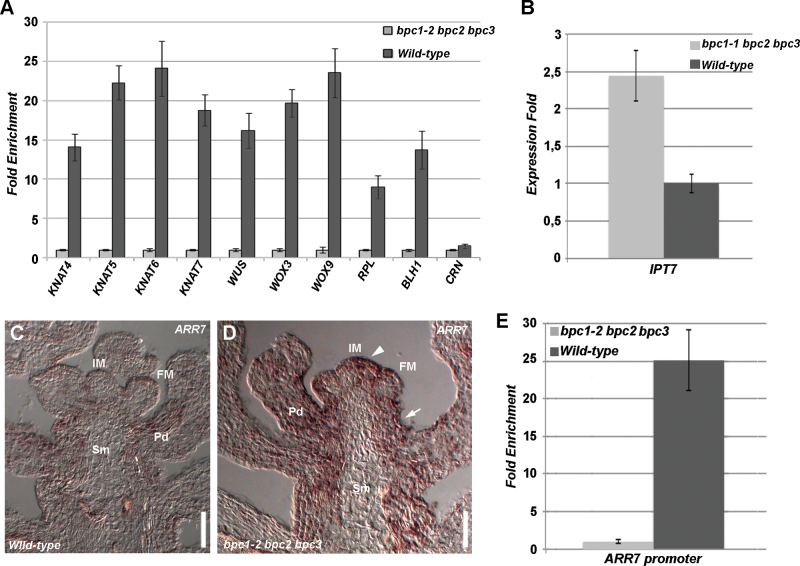
*HOMEOBOX* and cytokinin pathway genes are regulated by class I BPCs. (A) ChIP analysis revealing that class I BPCs directly bind the GA-rich sites in the promoter of different HOMEOBOX transcription factors; the *bpc1-2 bpc2 bpc3* triple mutant was used as a negative control. Each bar shows the average of three independent ChIP experiments (±standard deviation). (B) Expression analyses of *IPT7* in wild-type and *bpc1-2 bpc2 bpc3* young inflorescences. (C, D) *In situ* hybridization with *ARR7*-specific antisense probe using wild-type (C) and *bpc1-2 bpc2 bpc3* (D) inflorescences. A stronger and more diffuse signal was detectable in the mutant IM (arrowhead), FM, pedicels, and stem (arrow). (E) ChIP analysis revealing that class I BPCs directly bind the GA-rich site in the *ARR7* promoter; the *bpc1-2 bpc2 bpc3* triple mutant was used as a negative control. Each bar shows the average of three independent ChIP experiments (±standard deviation). Pd, pedicel; Sm, stem. Bars, 50 µm. (This figure is available in colour at *JXB* online.)

Analysis of the putative promoter regions of other HOMEOBOX transcription factor-encoding genes belonging to different families showed that 53 out of 88 genes that we analysed contained one or more GA-rich repeats that were similar to those observed in the *STM* promoter (Supplementary Table S1 available at *JXB* online). We selected a few genes that were expressed in the *Arabidopsis* inflorescence, belonging to the *KNOX*, *BELL*, *WUS*, and *HD-ZIP* families, and that contained GA-rich sequences 500bp upstream of their transcription start site. Using ChIP assays, we verified whether class I BPCs directly bound them. The genes that we selected were *KNAT4*, *KNAT5*, *KNAT6*, and *KNAT7* from the *KNOX* family; *WUS*, *WUSCHEL RELATED HOMEOBOX 3* (*WOX3*), and *WOX9* belonging to the *WUS* family; *REPLUMLESS* (*RPL*) and *BELL-LIKE HOMEOBOX1* (*BLH1*) belonging to the *BELL* family; and *CORONA* (*CRN*) from the *HD-ZIP* family. Except for *CRN*, the GA-rich sequences located in the putative promoter regions of all these genes was shown to be highly enriched in three independent ChIP experiments (Fig. 4A), suggesting that they are all direct targets of class I BPC factors.

### The CK pathway is upregulated in the *bpc1-2 bpc2 bpc3* triple mutant

CKs form a class of plant hormones involved in many aspects of plant development, such as shoot and root meristem formation and activity (Werner *et al.*, 2003), vascular tissue formation (Mähönen *et al.*, 2000), apical dominance, leaf senescence, cell differentiation (Dello Ioio *et al.*, 2007), and cell division (Dewitte *et al.*, 2007). Mutants with overproduction or slow degradation of CKs display enlarged IMs and extrafloral organs (Venglat and Sawhney, 1996; Bartrina *et al.*, 2011; Bencivenga *et al.*, 2012). On the other hand, in mutants with impaired CK biosynthesis or CK perception, the meristem differentiates and terminates prematurely (Bartrina *et al.*, 2011).

A few *KNOX* genes such as *STM* and *BP* are known to be involved in the CK pathway (Venglat *et al.*, 2002; Yanai *et al.*, 2005; Jasinski *et al.*, 2005; Leibfried *et al.*, 2005; Bartrina *et al.*, 2011; Scofield *et al.*, 2013) by directly activating transcription of the *ISOPENTENYLTRANSFERASE 7* (*IPT7*) gene, which encodes a key enzyme involved in the CK synthesis pathway (Kakimoto, 2001). Subsequently, CK signalling is propagated through a set of more than 20 response regulators (ARRs; Heyl *et al.*, 2008) and one of the final goals is the activation of *WUS* in the meristem to promote the maintenance of meristematic tissue (Jasinski *et al.*, 2005; Leibfried *et al.*, 2005).

The enlarged IM and the increase in *STM* and *BP* expression levels as observed in the *bpc1-2 bpc2 bpc3* triple mutant were in agreement with a possible increase in the CK content at the IM. To support this hypothesis, we investigated by qRT-PCR the *IPT7* expression levels in wild-type and *bpc1-2 bpc2 bpc3* inflorescences (meristem and young floral buds; Fig. 4B). This revealed that the expression level of *IPT7* was significantly higher in the *bpc1-2 bpc2 bpc3* mutant, which was in accordance with the previously detected overexpression of *STM*. To further support the hypothesis of an increase in CK concentration in the triple *bpc* mutant meristem, the expression pattern of the *ARABIDOPSIS RESPONSE REGULATOR 7* (*ARR7*) gene was investigated by *in situ* hybridization using a specific *ARR7* probe (Buechel *et al.*, 2010). *ARR7* is a primary CK response gene and is rapidly upregulated by exogenous CK application (Buechel *et al.*, 2010; Zhao *et al.*, 2010). This revealed that, in the wild-type IM, *ARR7* mRNA levels were rather low (Fig. 4C; Buechel *et al.*, 2010; Zhao *et al.*, 2010), whereas in the *bpc1-2 bpc2 bpc3* triple mutant, the *ARR7* hybridization signal was stronger (Fig. 4D), suggesting that CK-mediated signalling was more active in the IM. Interestingly, a palindromic GAGA box localized in the *ARR7* promoter at –178bp from the transcription start site (Supplementary Fig. S4 available at *JXB* online) was highly enriched when tested in three independent ChIP experiments using antibodies against class I BPCs (Fig. 4E). This strongly supports an involvement of class I BPCs at multiple levels in regulation of the CK pathway in the meristem.

## Discussion

The functional characterization of *BPC* genes has only recently been initiated (Meister *et al.*, 2004; Monfared *et al.*, 2011: Simonini *et al.*, 2012). Interestingly, all these genes are widely expressed throughout the plant, and higher-order *bpc* mutant combinations have shown developmental defects in both vegetative and reproductive tissues (Monfared *et al.*, 2011). These studies clearly revealed that they act redundantly during plant development. Previously, it was shown that the *bpc1-1 bpc2* double mutant had a more severe phenotype than the *bpc1-1 bpc2 bpc3* triple mutant (Monfared *et al.*, 2011), suggesting that the loss of *BPC3* activity compensates for the loss of *BPC1* and *BPC2*. Under our greenhouse conditions, this observation was not observed with respect to the meristem defects that we described here. These defects were only observed in the *bpc1-2 bpc2 bpc3* triple mutant and not in the *bpc1-2 bpc2* double mutant. However, in our study, we used for all our analyses the *bpc1-2* complete knockout allele (Simonini *et al.*, 2012), and, indeed, in the *bpc1-1 bpc2 bpc3* triple mutant, such meristem defects were more rare and milder, suggesting that complete loss of BPC1 activity is needed to observe the meristem phenotypes that we described here.

The class I BPC factors seem to directly regulate *HOMEOBOX* genes of different classes. The fact that many *HOMEOBOX* genes controlling meristem functions are directly bound by class I BPC proteins underlines their potential importance in the control of plant development. Considering that the antibodies specifically recognize the class I BPC proteins (Simonini *et al.*, 2012), we can, of course, not exclude that also BPCs of other classes are involved in the regulation of these genes. However, as the *bpc1-2 bpc2 bpc3* mutant has larger IMs and FMs (whereas for instance the *bpc4 bpc6* double mutant does not have this phenotype), it is clear that class I BPCs at least are important for the regulation of genes controlling meristem size.

*STM* is a key gene for meristem tissue maintenance and is strongly expressed in both vegetative and reproductive meristems. Loss-of-function alleles of *STM* display precocious deprivation of meristem tissue in the IM. Therefore, these plants produce only a few flowers with fewer floral organs (Durbak and Tax, 2011). In contrast, upregulation of *STM* expression leads to an IM enlargement connected to an increase in meristem activity (Yanai *et al.*, 2005). *STM* is involved in the CK pathway, a class of hormones tightly linked to meristem activity; indeed, loss of meristem function in the *stm* mutant can be rescued by exogenous CK application or by the expression of a CK biosynthetic gene driven by the *STM* promoter (Yanai *et al.* 2005).

*STM* is upregulated in the *bpc1-2 bpc2 bpc3* background, and this is consistent with enlargement of the IM detected in this mutant. Moreover, this regulation seems to be direct, as BPCs of class I strongly bind the *STM* promoter.

As *STM* is responsible for CK synthesis in the meristem (Yanai *et al.*, 2005), we investigated whether CK levels were altered in the *bpc1-2 bpc2 bpc3* triple mutant by checking the expression profiles of *pCLV3:GUS* (Gross-Hardt *et al.*, 2002), *WUS* (Mayer *et al.*, 1998), and *ARR7* (Buechel *et al.*, 2010; Zhao *et al.*, 2010).

The *pCLV3::GUS* expression domain, which is not only a marker for meristem size but is also indicative of CK signalling (Gordon *et al.*, 2009), is expanded in the *bpc1-2 bpc2 bpc3* triple mutant IM. This expansion of the *pCLV3::GUS* domain could be a consequence of the meristem enlargement but could also be a response to the increment in CK content, as exogenous CK treatment stimulates the expansion of *pCLV3::GFP–EAR* reporter gene expression within IMs and FMs (Gordon *et al.*, 2009).

*WUS*, which promotes meristem proliferation (Laux *et al.*, 1996) and which is positively regulated by CK (Gordon *et al.*, 2009), did not significantly expand its expression domain in the *bpc1-2 bpc2 bpc3* background. This seems to be in contrast with the enlarged meristem and expanded *CLV3* domain. However, expansion of the *CLV3* expression domain is not always correlated with an expansion in *WUS* expression, suggesting that the feedback loop that regulates *WUS* expression in the meristem could occur through both CLAVATA-dependent and -independent pathways (Gordon *et al.*, 2009; Yoshida *et al.*, 2011). This could be the case for the *bpc1-2 bpc2 bpc3* mutant, in which the expansion of the *CLV3* domain did not seem to be accompanied by a *WUS* domain expansion. Thus, expansion of the *CLV3* expression domain might be more a consequence of the increased meristem size rather than being caused directly by the loss of BPC protein activities. The fact that *WUS* seems to be a direct target of BPCs (whereas *CLV3* does not have BPC-binding sites in its genomic region and therefore is probably not a direct target of BPCs) might place *CLV3* and *WUS* in two different pathways.

The expression levels of both *IPT7* and *ARR7*, which are a CK biosynthetic and a CK responsive gene, respectively (Kakimoto, 2001; Buechel *et al.*, 2010; Zhao *et al.*, 2010), were upregulated in the *bpc1-2 bpc2 bpc3* triple mutant, suggesting that, in this mutant, CK levels are increased. Moreover, *ARR7* is a direct target of BPCs, strengthening their direct role in regulation of the CK pathway at multiple levels in the meristem.

These data therefore support the hypothesis that, in the *bpc1-2 bpc2 bpc3* triple mutant IM, the activity is higher due to increased production of CK, which is probably caused by the upregulation of *KNOXI* genes like *STM* and *BP* (Jasinski *et al.*, 2005; Yanai *et al.*, 2005: Sakamoto *et al.*, 2006). Indeed, in the *35S::STM::GR* inducible line, the levels of several CKs increased within 24h of induction (Yanai *et al.*, 2005); moreover, an expansion of the *ARR5* expression domain is observed in *35S::BP* lines, where *BP* is constitutively misexpressed in leaves (Yanai *et al.*, 2005).

*BP* and *RPL* are two other HOMEOBOX transcription factors to which BPCs of class I directly bind. Both genes are involved in stem elongation and in inflorescence architecture (Ori *et al.*, 2000; Venglat *et al.*, 2002; Smith and Hake, 2003; Kanrar *et al.*, 2008), and their repression could be responsible for the inability of the 35S::BPC1–EAR motif plants to produce a stem and a well-organized inflorescence. The loss of the spiral pattern in the *bpc1-2 bpc2 bpc3* inflorescence is reminiscent of *rpl* mutant plants, and it will be interesting to investigate whether this gene is regulated by BPCs.

BPC factors form a plant-specific transcription factor family. However, despite the fact that their amino acid sequence seems to be unrelated to animal GAGA-binding proteins, they have been suggested to play similar roles in plants (Berger and Dubreucq, 2012; Simonini *et al.*, 2012). The analysis that we have described here points again to an evolutionary relationship between the animal and plant GAGA-binding proteins. In *Drosophila*, the GAGA factor (dGAF) has been shown to be important in particular for the regulation of *HOMEOBOX* genes and in this way controlling a wide range of developmental events (Botas, 1993; Graba *et al.*, 1997). The fact that GAGA-binding proteins of animals and plants are important in controlling the activity of *HOMEOBOX* genes might, of course, be a coincidence, but it remains an interesting parallel between these ‘unrelated’ factors.

The molecular mechanisms by which BPC proteins regulate their target genes are not yet clear. However, recently we showed that they loop the promoter region of the ovule identity gene *STK* and that they interact with a MADS-domain protein containing repressor complex to silence *STK* expression in the FM (Kooiker *et al.*, 2005; Gregis *et al.*, 2006; Simonini *et al.*, 2012). It is likely that BPC proteins interact with transcription factor complexes to facilitate their binding to the DNA. Therefore, also in the case of genes like *STM* and *BP*, it might well be that BPCs interact with the upstream regulators to recruit them to the promoters. ASYMMETRIC LEAVES1 (AS1) and AS2 are known to directly repress *STM* expression in leaves (Uchida *et al.*, 2007). It will be interesting to verify whether BPCs interact with AS1 and AS2. In the *bpc1-2 bpc2 bpc3* triple mutant, we observed mainly an upregulation of *STM* in the meristem and flowers, and no ectopic expression in leaves. It seems, therefore, that for the regulation of *STM* and *BP* in these tissues, the class I BPC proteins have more of a role in fine-tuning the expression of these genes, rather than acting as the main regulators.

A similar observation was shown for the *STK* gene (Simonini *et al.*, 2012). When the BPC binding sites were mutated in the *STK* promoter, its expression, which is normally active only in developing ovules, was completely deregulated, and promoter activity was observed throughout the flower. However, also in this case, no expression was observed in the vegetative parts of the plant. It might be that the repression mechanisms facilitated by BPCs are different between reproductive and vegetative tissues. Further investigations will be needed to obtain a better understanding of these molecular mechanisms.

## Supplementary data

Supplementary data are available at *JXB* online.

Supplementary Fig. S1. Angle divergence in the *bpc1-2 bpc2 bpc3* mutant.

Supplementary Fig. S2. Expression level of *WUS* in *bpc1-2 bpc2 bpc3* mutant.

Supplementary Fig. S3. Expression level of the *BPC1–EAR* chimeric gene in plants with a strong phenotype.

Supplementary Fig. S4. Localization of GAGA boxes in *STM*, *BP*, and *ARR7* promoters.

Supplementary Table S1. *HOMEOBOX* genes with a GAGA stretch in their promoter sequence (500bp upstream of the transcription start site).

Supplementary Table S2. Primers used in this study.

Supplementary Data

## Abbreviations:

ChIP: chomatin immunoprecipitation
CK: cytokinin
DAPI: 4′,6-diamidino-2-phenylindole
FM: floral meristem
GUS: β-glucuronidase
IM: inflorescence meristem
mPS-PI: modified pseudo-Schiff propidium iodide
qRT-PCR: quantitative real-time PCR
SEM: scanning electron microscopy.
