# Supplementary material for: Class I BASIC PENTACYSTEINE factors regulate HOMEOBOX genes involved in meristem size maintenance
Source: J Exp Bot. 2014 Jan 30;65(6):1455–65. doi: 10.1093/jxb/eru003 (PMC3967085; doi:10.1093/jxb/eru003)
Supplement: Supplementary Data [file supp_65_6_1455__index.html]

Class I BASIC PENTACYSTEINE factors regulate HOMEOBOX genes involved in meristem size maintenance — Class I BASIC PENTACYSTEINE factors regulate HOMEOBOX genes involved in meristem size maintenance — Supplementary Data 

# Class I BASIC PENTACYSTEINE factors regulate *HOMEOBOX* genes involved in meristem size maintenance

## Supplementary Data

Data files

**Files in this Data Supplement:**

- Supplementary Data - Supplementary Data
