# Supplementary material for: Class I BASIC PENTACYSTEINE factors regulate HOMEOBOX genes involved in meristem size maintenance
Source: J Exp Bot. 2014 Jan 30;65(6):1455–65. doi: 10.1093/jxb/eru003 (PMC3967085; doi:10.1093/jxb/eru003)
Supplement: Supplementary Data [file supp_eru003_jexbot107656_file001.pdf]

Supplementary Figure 1

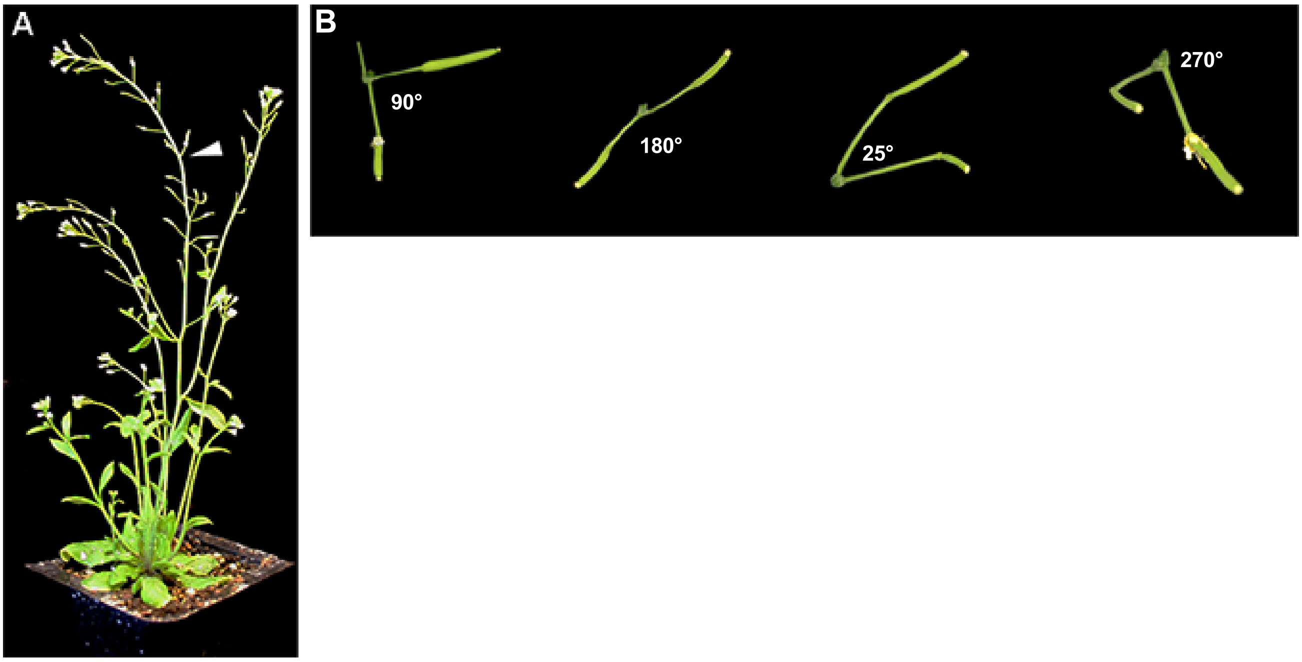

Supplememtary Figure 1. Angle divergence in *bpc1-2 bpc2 bpc3* mutant

(A) *bpc1-2 bpc2 bpc3* plant. Siliques are randomly positioned along the stem and two siliques arising from the same internode can be frequently observed (arrowhead).

(B) Four examples of divergence angles frequently found in the *bpc1-2 bpc2 bpc3* mutant: from left to right 90°, 180°, 25°, 270°.

Supplementary Figure 2.

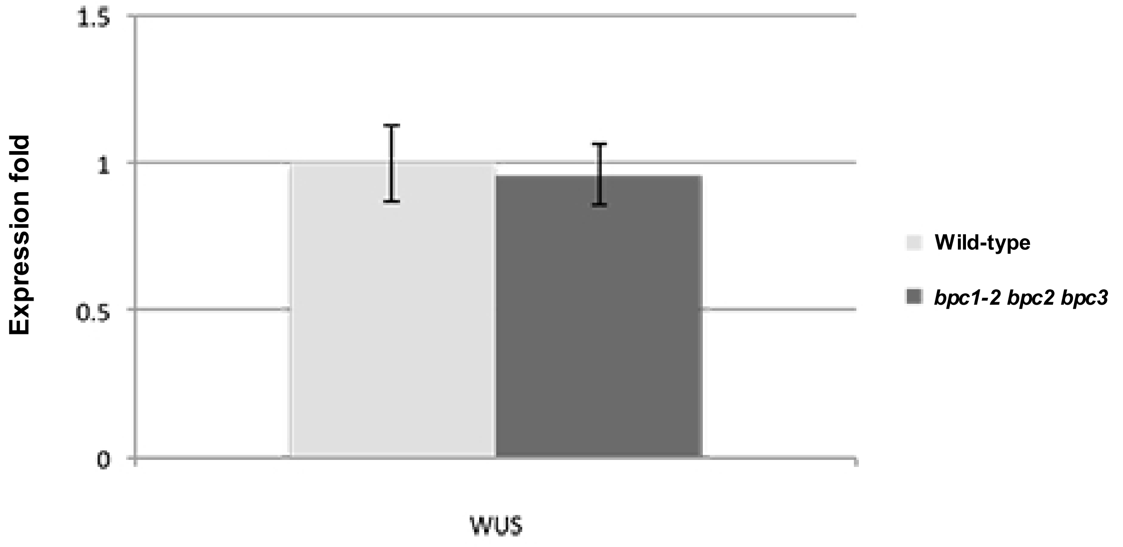

Supplementary Figure 2. Expression level of *WUS* in *bpc1-2 bpc2 bpc3* mutant.

Supplementary Figure 3

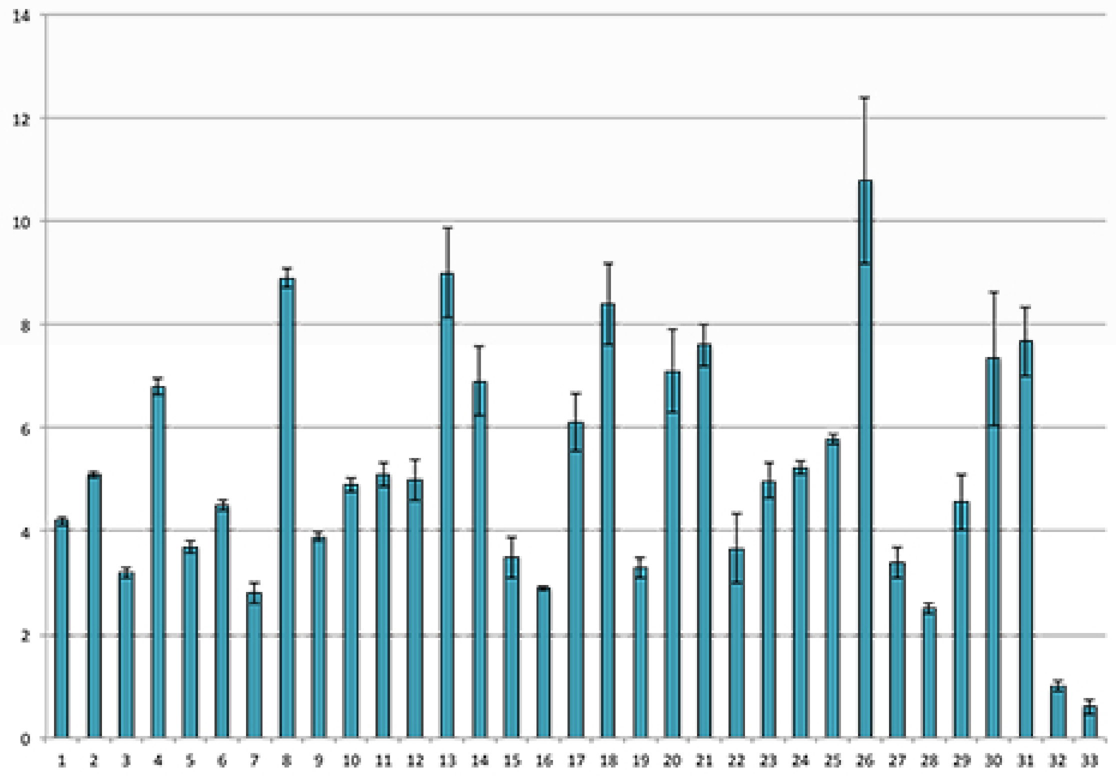

Supplementary Figure 3. Expression level of BPC1-Ear Motif in plants with strong phenotype.
From number 1 to number 31: *35S::BPC1 Ear Motif* plants with phenotype.
Numbers 32 and 33: *35S::BPC1 Ear Motif* plants with wild-tpe phenotype.

Supplementary Figure 4

STM promoter

.....taataatactagtgagagaaagagaagATGGAGAGTGGTTCCAACA....

BP promoter

acagaagagagaagcctttgccttatctcttgtcccttctctcttacctttattttaattttcaaata
tttctttttgctcccaaagcaaacgacgtcttgtcaatccactcaagccacccaacttcttcatt
attgttaatctctctctctctctctttctctcttcttctctctttctcttttttttttttttattttcttctcttcca
tgtcacttccttgacgaattctatatacctagttcgtttttcttcctcaaatatatctttttcaatttatt
tggtttttctttgggtgcaacttcacctcacaaaattttctctcttttttatattaatttgagttaggcc
tttttgatttcatagatgagtcgtctagtcgtctggatttgatgtggttatagtcttacagagaccttt
gattgaaataagaacaaaagcaagaatacatacatcctcttcatcttacacccatcctttttta
tttttctagggttttatttttttttaatttattttttttctttgatttttatattctctctctctctcaaatctttact
catctgggtATGGAAGAATACCAGCATGACA

ARR7 promoter

aatcttaaaaaagattcggtgcccaaagattttgacctttgaagacacatttgattatttgtgtttt
ctgacacttcccaaacacataacatcaacacttgacccatttttaagattttgtttcctcttcaaa
gatcttaccccgcaagatccgatcttccaaatctctctctatctactatactagtacttattaaac
cccaccacctctcccttcttatttatgtcacctaacctaacttcttcttctttcattttatcacattca
tttcctttgtcttcaatccaaagctttcctcttcttggaaccaatctgctctctttttttattctgagttt
gacaATGGCGGTTGGTGAGGTCATGAG

Supplementary Figure 4.
Localization of GAGA boxes in *STM*, *BP* and *ARR7* promoters.
Red box: transcription start site
Blue box: GAGA box 5’ -3’
Green box: BABA box 3’-5’

Supplementary table 1. HOMEOBOX genes with GAGA stretch in their promoter sequence (500bp upstreamt the transcription start site). Green box: at least one GAGA stretch found. Red box: no GAGA sequence found.

Locus	Gene Name	GAGA
At4g00730	ANL2	Green
At4g32980	ATH1	Red
At3g01470	Athb-1	Green
At1g79840	Athb-10	Green
At3g61890	Athb-12	Green
At1g69780	Athb-13	Green
At2g34710	PHB	Green
At1g52150	CNA	Green
At4g40060	Athb-16	Green
At2g01430	Athb-17	Green
At4g16780	Athb-2	Red
At5g15150	Athb-3	Green
At2g44910	Athb-4	Green
At5g65310	Athb-5	Green
At2g22430	Athb-6	Green
At2g46680	Athb-7	Red
At4g32880	Athb-8	Green
At1g30490	PHV	Green
At4g21750	ATML1	Green
At5g41410	BEL1	Green
At4g36870	BLH2	Green
At1g75410	BLH3	Green
At2g23760	BLH4	Green
At1g19700	BLH5	Red
At5g02030	BLR	Green
At4g25530	FWA	Red
At4g17460	HAT1	Red
At5g06710	HAT14	Red
At5g47370	HAT2	Green
At4g37790	HAT22	Green
At3g60390	HAT3	Red
At3g19510	HAT3.1	Green
At2g22800	HAT9	Red
At3g61150	HD-GL2-1	Green
At4g08150	KNAT1 (BP)	Green
At1g70510	KNAT2	Green
At5g25220	KNAT3	Green
At5g11060	KNAT4	Green
At4g32040	KNAT5	Green
At1g23380	KNAT6	Green
At1g62990	KNAT7	Green
At4g02560	LD	Green
At4g17710	HDG4	Red
At1g26960	ATHB23	Red
At3g01220	ATHB20	Green
At1g17920	HDG12	Green
At1g73360	HDG11	Green
At1g27050	ATHB54	Red
At1g70920	ATHB18	Green
At1g05230	HDG2	Green
At1g34650	HDG10	Red
At2g18550	ATHB21	Green
At2g32370	HDG3	Red
At2g36610	ATHB22	Red
At3g03260	HDG8	Green
At4g36740	ATHB40	Red
At5g17320	HDG9	Red
At5g52170	HDG7	Red
At5g03790	ATHB51	Red
At5g66700	ATHB53	Red
At5g46880	HDG5	Green
At1g28420	HB-1	Red
At1g75430	BLH11	Green
At2g16400	BLH7	Red
At2g27220	BLH5	Red
At2g35940	BLH1	Green
At4g34610	BLH6	Red
At5g44180		Red
At5g46010		Red
At4g03250		Red
At5g53980	ATHB52	Red
At4g04890	PDF2	Green
At2g27990	POUND-FOOLISH	Red
At4g29940	PRHA	Green
At5g60690	REV	Green
At1g62360	STM	Green
At3g18010	WOX1	Green
At1g20710	WOX10/WOX13B	Green
At3g03660	WOX11	Red
At5g17810	WOX12	Red
At4g35550	WOX13	Red
At1g20700	WOX14	Red
At5g59340	WOX2	Green
At2g28610	WOX3	Green
At1g46480	WOX4	Red
At3g11260	WOX5	Red
At2g01500	WOX6	Red
At5g05770	WOX7	Red
At5g45980	WOX8	Red
At2g33880	WOX9	Green

Supplementary table 2. Primer used in this manuscript.

Gene	Primer sequences
BPC1-EAR motif **construct**	**Fw-** CACCATGGACGATGATGGATTTCGC<br>**Rev-** CTAAGCAAATCCAAGTCTAAGTTCAAGATC AAGATCAAGTCTGATCGTGACAAACTTATTGG
BPC1-EAR motif **expression**	**Fw-** GCTCTTGTTTTTCTTCATTGATCG<br>**Rev-** CAAGATCAAGATCAAGTCTGATCGTGACAAACTTATTGG
KNAT4 **promoter**	**Fw-** CGTCAAACACACATACACATCG<br>**Rev-** GATGTTGTTGTTGGTCGGTG
KNAT5 **promoter**	**Fw-** ACTCTCCCGTCACATTCTGG<br>**Rev-** GTCTTGGATTAGTTGAGCGG
KNAT6 **promoter**	**Fw-** CGACCTATGAAGCCTGAAGC<br>**Rev-** CTCCTCCGTCTTCTAAATCGC
KNAT7 **promoter**	**Fw-** GGACCAGACCCGTGTATAAC<br>**Rev-** TACCTAGTGCCGCTTCTTGC
BP **promoter**	**Fw-** TCAATCCACTCAAGCCACCC<br>**Rev-** TCGTCAAGGAAGTGACATGG
BP **expression**	**Fw-** CACCGTCTGTCTCTGCCTCC<br>**Rev-** TCCCCTCCGCTGTTATTCTC
STM **promoter**	**Fw-** CTTACTCTTTTAGGGTTTCC<br>**Rev-** ATCATAGGACACATCGGACC
STM **expression**	**Fw-** CCTCACCTTCCTCTTTCTCC<br>**Rev-** TGTTGACGAGCTTCTTTAGG
WUS **promoter**	**Fw-** AAGCACATTTTTCAATAGGG<br>**Rev-** CGGCTTGATGATGATGATGC
WUS **expression**	***Gregis et al., 2013***
WOX3 **promoter**	**Fw-** TGGGAGCATATGAGATTGGG<br>**Rev-** CGGCTTGATGATGATGATGC
WOX9 **promoter**	**Fw-** ATGAAAACAGGTTGGGTAGG<br>**Rev-** AGAGACAGGGAACAGAATGG
RPL **promoter**	**Fw-** TGCTCATACTAAATTCCTCC<br>**Rev-** GGATGATGTCGATGAGATGC
BLH1 **promoter**	**Fw-** GGCGTACTAAGGACTGTGGG<br>**Rev-** ATCATCTCAAGCTGCAAAAGG
CRN **promoter**	**Fw-** CTTTTACAGGCATTATTGGG<br>**Rev-** GGAATGGGGAAGGTCAGAGG
ARR7 **promoter**	**Fw-** AAAGTTCCAGGTCACGAGGG<br>**Rev-** AGGTGGTGGGGTTTAATAAG
IPT7 **expression**	***Takano et al., 2010***
